# Supplementary material for: Modeling glioblastoma heterogeneity as a dynamic network of cell states
Source: Mol Syst Biol. 2021 Sep 16;17(9):e10105. doi: 10.15252/msb.202010105 (PMC8444284; doi:10.15252/msb.202010105)
Supplement: Supplementary file 6 — Source Data for Figure 5 [file MSB-17-e10105-s004.zip › Figure5A_sourcedata/GSEA_3071/nosigdb_stateIII.GseaPreranked.1622795155562/RICHARDS_NOWAKOWSKI_SCIENCE_2017_ASTROCYTE_UPREG.html]

Details for gene set RICHARDS\_NOWAKOWSKI\_SCIENCE\_2017\_ASTROCYTE\_UPREG[GSEA]

|  || Dataset | state33071 |
| Phenotype | NoPhenotypeAvailable |
| Upregulated in class | na\_neg |
| GeneSet | RICHARDS\_NOWAKOWSKI\_SCIENCE\_2017\_ASTROCYTE\_UPREG |
| Enrichment Score (ES) | -0.41013867 |
| Normalized Enrichment Score (NES) | -2.8592613 |
| Nominal p-value | 0.0 |
| FDR q-value | 0.0 |
| FWER p-Value | 0.0 |
Table: GSEA Results Summary

  

Fig 1: Enrichment plot: RICHARDS\_NOWAKOWSKI\_SCIENCE\_2017\_ASTROCYTE\_UPREG      
 Profile of the Running ES Score & Positions of GeneSet Members on the Rank Ordered List

  

| PROBE | GENE SYMBOL | GENE\_TITLE | RANK IN GENE LIST | RANK METRIC SCORE | RUNNING ES | CORE ENRICHMENT || 1 | RAB13 |  |  | 18 | 0.539 | -0.0006 | No |
| 2 | MT2A |  |  | 31 | 0.499 | 0.0000 | No |
| 3 | POLR2L |  |  | 38 | 0.482 | 0.0022 | No |
| 4 | RHOC |  |  | 92 | 0.414 | -0.0089 | No |
| 5 | GNG5 |  |  | 139 | 0.382 | -0.0185 | No |
| 6 | NDUFA4 |  |  | 140 | 0.382 | -0.0154 | No |
| 7 | SPATS2L |  |  | 184 | 0.351 | -0.0244 | No |
| 8 | PRDX1 |  |  | 220 | 0.328 | -0.0313 | No |
| 9 | LGALS1 |  |  | 230 | 0.323 | -0.0312 | No |
| 10 | ENO1 |  |  | 241 | 0.314 | -0.0314 | No |
| 11 | TOMM7 |  |  | 264 | 0.303 | -0.0350 | No |
| 12 | SEPT7 |  |  | 267 | 0.303 | -0.0332 | No |
| 13 | PSME2 |  |  | 310 | 0.289 | -0.0423 | No |
| 14 | NDUFB3 |  |  | 312 | 0.288 | -0.0403 | No |
| 15 | MRPS5 |  |  | 342 | 0.280 | -0.0460 | No |
| 16 | EFHD2 |  |  | 383 | 0.266 | -0.0548 | No |
| 17 | MRFAP1 |  |  | 404 | 0.259 | -0.0582 | No |
| 18 | APC |  |  | 467 | -0.253 | -0.0731 | No |
| 19 | ST3GAL5 |  |  | 519 | -0.255 | -0.0850 | No |
| 20 | PLOD2 |  |  | 521 | -0.255 | -0.0833 | No |
| 21 | HIPK2 |  |  | 546 | -0.257 | -0.0878 | No |
| 22 | PTPRA |  |  | 552 | -0.257 | -0.0871 | No |
| 23 | NTM |  |  | 553 | -0.257 | -0.0851 | No |
| 24 | TMEM132B |  |  | 578 | -0.258 | -0.0896 | No |
| 25 | LITAF |  |  | 582 | -0.259 | -0.0883 | No |
| 26 | ZNF236 |  |  | 592 | -0.259 | -0.0887 | No |
| 27 | PTPN13 |  |  | 622 | -0.261 | -0.0946 | No |
| 28 | MLEC |  |  | 627 | -0.261 | -0.0936 | No |
| 29 | AGPAT3 |  |  | 665 | -0.263 | -0.1016 | No |
| 30 | ZFP36L2 |  |  | 671 | -0.263 | -0.1009 | No |
| 31 | EEPD1 |  |  | 685 | -0.264 | -0.1024 | No |
| 32 | TRPS1 |  |  | 690 | -0.264 | -0.1014 | No |
| 33 | LDLRAD3 |  |  | 698 | -0.264 | -0.1012 | No |
| 34 | LINC01138 |  |  | 747 | -0.267 | -0.1121 | No |
| 35 | SC5D |  |  | 756 | -0.267 | -0.1122 | No |
| 36 | PBXIP1 |  |  | 771 | -0.268 | -0.1139 | No |
| 37 | MFGE8 |  |  | 794 | -0.269 | -0.1178 | No |
| 38 | ETV5 |  |  | 818 | -0.270 | -0.1219 | No |
| 39 | LRRTM3 |  |  | 827 | -0.271 | -0.1219 | No |
| 40 | TAPBP |  |  | 846 | -0.272 | -0.1247 | No |
| 41 | SLC25A53 |  |  | 862 | -0.272 | -0.1266 | No |
| 42 | BCHE |  |  | 867 | -0.272 | -0.1256 | No |
| 43 | KAT2B |  |  | 877 | -0.273 | -0.1258 | No |
| 44 | IQSEC1 |  |  | 900 | -0.274 | -0.1297 | No |
| 45 | CRYL1 |  |  | 939 | -0.276 | -0.1378 | No |
| 46 | PMP22 |  |  | 1023 | -0.281 | -0.1583 | No |
| 47 | SALL2 |  |  | 1031 | -0.281 | -0.1579 | No |
| 48 | CPE |  |  | 1081 | -0.284 | -0.1691 | No |
| 49 | PHYHIPL |  |  | 1087 | -0.284 | -0.1682 | No |
| 50 | LPP |  |  | 1097 | -0.284 | -0.1684 | No |
| 51 | GPC6 |  |  | 1101 | -0.285 | -0.1669 | No |
| 52 | CERS6 |  |  | 1140 | -0.287 | -0.1750 | No |
| 53 | TFPI |  |  | 1141 | -0.287 | -0.1727 | No |
| 54 | KAT2A |  |  | 1192 | -0.290 | -0.1841 | No |
| 55 | ZMYM2 |  |  | 1207 | -0.291 | -0.1856 | No |
| 56 | SPATA6 |  |  | 1225 | -0.291 | -0.1879 | No |
| 57 | METTL7A |  |  | 1280 | -0.294 | -0.2003 | No |
| 58 | PLEKHH2 |  |  | 1282 | -0.294 | -0.1983 | No |
| 59 | CTNND2 |  |  | 1304 | -0.295 | -0.2017 | No |
| 60 | PRNP |  |  | 1309 | -0.295 | -0.2004 | No |
| 61 | PHLPP1 |  |  | 1317 | -0.296 | -0.2000 | No |
| 62 | FUT11 |  |  | 1324 | -0.296 | -0.1993 | No |
| 63 | GSTM4 |  |  | 1374 | -0.299 | -0.2103 | No |
| 64 | BBS2 |  |  | 1404 | -0.300 | -0.2158 | No |
| 65 | LRP1B |  |  | 1427 | -0.302 | -0.2194 | No |
| 66 | FADS2 |  |  | 1437 | -0.302 | -0.2195 | No |
| 67 | LRP4 |  |  | 1454 | -0.303 | -0.2214 | No |
| 68 | MTSS1L |  |  | 1479 | -0.305 | -0.2255 | No |
| 69 | GRIA4 |  |  | 1488 | -0.305 | -0.2253 | No |
| 70 | ETV1 |  |  | 1495 | -0.306 | -0.2245 | No |
| 71 | TGIF1 |  |  | 1506 | -0.306 | -0.2248 | No |
| 72 | RNF13 |  |  | 1521 | -0.307 | -0.2262 | No |
| 73 | ROBO3 |  |  | 1527 | -0.308 | -0.2251 | No |
| 74 | MTRNR2L8 |  |  | 1536 | -0.308 | -0.2249 | No |
| 75 | OLFM2 |  |  | 1548 | -0.309 | -0.2254 | No |
| 76 | AHCYL2 |  |  | 1562 | -0.310 | -0.2265 | No |
| 77 | ZEB1 |  |  | 1569 | -0.310 | -0.2257 | No |
| 78 | SPRY2 |  |  | 1592 | -0.311 | -0.2292 | No |
| 79 | DAG1 |  |  | 1598 | -0.312 | -0.2281 | No |
| 80 | MAML2 |  |  | 1603 | -0.312 | -0.2267 | No |
| 81 | ALDOC |  |  | 1613 | -0.313 | -0.2267 | No |
| 82 | ADCY2 |  |  | 1640 | -0.315 | -0.2313 | No |
| 83 | C3orf70 |  |  | 1698 | -0.318 | -0.2443 | No |
| 84 | SLITRK2 |  |  | 1725 | -0.320 | -0.2489 | No |
| 85 | SCD |  |  | 1737 | -0.321 | -0.2493 | No |
| 86 | ID2 |  |  | 1780 | -0.323 | -0.2582 | No |
| 87 | CPT1A |  |  | 1816 | -0.325 | -0.2652 | No |
| 88 | CLDN10 |  |  | 1822 | -0.325 | -0.2640 | No |
| 89 | CXXC5 |  |  | 1889 | -0.329 | -0.2794 | No |
| 90 | DPF3 |  |  | 1902 | -0.330 | -0.2800 | No |
| 91 | SESN3 |  |  | 1920 | -0.331 | -0.2820 | No |
| 92 | EFCAB2 |  |  | 1922 | -0.331 | -0.2797 | No |
| 93 | ABLIM1 |  |  | 1940 | -0.332 | -0.2817 | No |
| 94 | SLC2A1 |  |  | 1961 | -0.333 | -0.2845 | No |
| 95 | FAM84B |  |  | 1975 | -0.334 | -0.2854 | No |
| 96 | CSGALNACT1 |  |  | 1976 | -0.334 | -0.2828 | No |
| 97 | SLC16A1 |  |  | 2012 | -0.336 | -0.2896 | No |
| 98 | MYO1E |  |  | 2017 | -0.337 | -0.2881 | No |
| 99 | LYPD1 |  |  | 2023 | -0.337 | -0.2868 | No |
| 100 | NOTCH1 |  |  | 2024 | -0.337 | -0.2841 | No |
| 101 | TIMP1 |  |  | 2038 | -0.337 | -0.2850 | No |
| 102 | PCDH17 |  |  | 2043 | -0.338 | -0.2834 | No |
| 103 | HMGCS1 |  |  | 2060 | -0.339 | -0.2850 | No |
| 104 | ZIC1 |  |  | 2062 | -0.339 | -0.2826 | No |
| 105 | SIRPA |  |  | 2072 | -0.339 | -0.2824 | No |
| 106 | TMED10 |  |  | 2100 | -0.341 | -0.2871 | No |
| 107 | TRIB2 |  |  | 2105 | -0.342 | -0.2854 | No |
| 108 | NLGN1 |  |  | 2107 | -0.342 | -0.2830 | No |
| 109 | PPIB |  |  | 2111 | -0.342 | -0.2811 | No |
| 110 | SCD5 |  |  | 2128 | -0.344 | -0.2827 | No |
| 111 | LGALS3BP |  |  | 2152 | -0.345 | -0.2863 | No |
| 112 | TNFRSF19 |  |  | 2165 | -0.346 | -0.2868 | No |
| 113 | DSEL |  |  | 2180 | -0.347 | -0.2879 | No |
| 114 | CANX |  |  | 2209 | -0.349 | -0.2927 | No |
| 115 | SASH1 |  |  | 2231 | -0.351 | -0.2957 | No |
| 116 | PAQR8 |  |  | 2246 | -0.352 | -0.2967 | No |
| 117 | MAGI1 |  |  | 2340 | -0.361 | -0.3192 | No |
| 118 | GJA1 |  |  | 2362 | -0.362 | -0.3221 | No |
| 119 | SPECC1 |  |  | 2368 | -0.363 | -0.3205 | No |
| 120 | TTYH1 |  |  | 2397 | -0.365 | -0.3253 | No |
| 121 | PRCP |  |  | 2489 | -0.372 | -0.3472 | No |
| 122 | ABAT |  |  | 2521 | -0.375 | -0.3526 | No |
| 123 | HEY1 |  |  | 2593 | -0.381 | -0.3690 | No |
| 124 | ADAMTS6 |  |  | 2604 | -0.382 | -0.3687 | No |
| 125 | PNISR |  |  | 2607 | -0.383 | -0.3662 | No |
| 126 | P2RY1 |  |  | 2613 | -0.383 | -0.3645 | No |
| 127 | PAX6 |  |  | 2619 | -0.384 | -0.3629 | No |
| 128 | HSPA2 |  |  | 2626 | -0.384 | -0.3614 | No |
| 129 | ABHD4 |  |  | 2648 | -0.386 | -0.3641 | No |
| 130 | ARAP2 |  |  | 2658 | -0.387 | -0.3635 | No |
| 131 | ID1 |  |  | 2685 | -0.389 | -0.3675 | No |
| 132 | TACC1 |  |  | 2728 | -0.393 | -0.3758 | No |
| 133 | NOTCH3 |  |  | 2759 | -0.395 | -0.3809 | No |
| 134 | HSPA1A |  |  | 2774 | -0.396 | -0.3816 | No |
| 135 | PSAP |  |  | 2808 | -0.400 | -0.3874 | No |
| 136 | SEMA6D |  |  | 2812 | -0.400 | -0.3850 | No |
| 137 | SLC35A5 |  |  | 2846 | -0.403 | -0.3908 | No |
| 138 | ALCAM |  |  | 2853 | -0.403 | -0.3893 | No |
| 139 | NRBP2 |  |  | 2857 | -0.404 | -0.3869 | No |
| 140 | PDE4B |  |  | 2878 | -0.406 | -0.3891 | No |
| 141 | ELOVL2 |  |  | 2879 | -0.406 | -0.3859 | No |
| 142 | ITGA6 |  |  | 2899 | -0.408 | -0.3879 | No |
| 143 | SERPINH1 |  |  | 2921 | -0.410 | -0.3903 | No |
| 144 | AASS |  |  | 2929 | -0.412 | -0.3890 | No |
| 145 | JUN |  |  | 2935 | -0.413 | -0.3871 | No |
| 146 | ITGAV |  |  | 2942 | -0.414 | -0.3854 | No |
| 147 | CD81 |  |  | 2945 | -0.414 | -0.3827 | No |
| 148 | RHOBTB3 |  |  | 2955 | -0.415 | -0.3819 | No |
| 149 | ITGB8 |  |  | 3026 | -0.424 | -0.3976 | No |
| 150 | DCLK2 |  |  | 3039 | -0.425 | -0.3975 | No |
| 151 | SEMA6A |  |  | 3042 | -0.425 | -0.3947 | No |
| 152 | ZNF678 |  |  | 3044 | -0.425 | -0.3916 | No |
| 153 | CHPT1 |  |  | 3060 | -0.426 | -0.3923 | No |
| 154 | ZMAT1 |  |  | 3090 | -0.430 | -0.3968 | No |
| 155 | GPRC5B |  |  | 3092 | -0.430 | -0.3937 | No |
| 156 | SLC48A1 |  |  | 3094 | -0.430 | -0.3905 | No |
| 157 | PRKD1 |  |  | 3146 | -0.437 | -0.4010 | No |
| 158 | GPM6A |  |  | 3171 | -0.440 | -0.4040 | No |
| 159 | SPON1 |  |  | 3187 | -0.441 | -0.4046 | No |
| 160 | MYO10 |  |  | 3196 | -0.442 | -0.4033 | No |
| 161 | PCDHGC3 |  |  | 3213 | -0.444 | -0.4041 | No |
| 162 | WSB1 |  |  | 3218 | -0.445 | -0.4017 | No |
| 163 | GABBR1 |  |  | 3226 | -0.447 | -0.4001 | No |
| 164 | SEMA5A |  |  | 3229 | -0.447 | -0.3971 | No |
| 165 | MSMO1 |  |  | 3241 | -0.450 | -0.3965 | No |
| 166 | HTRA1 |  |  | 3242 | -0.450 | -0.3929 | No |
| 167 | PTPRG |  |  | 3246 | -0.451 | -0.3902 | No |
| 168 | CD63 |  |  | 3258 | -0.452 | -0.3896 | No |
| 169 | ARHGEF26 |  |  | 3304 | -0.461 | -0.3982 | No |
| 170 | CDCA7L |  |  | 3311 | -0.462 | -0.3962 | No |
| 171 | ITM2B |  |  | 3314 | -0.463 | -0.3930 | No |
| 172 | MEST |  |  | 3363 | -0.473 | -0.4024 | No |
| 173 | NCKAP5 |  |  | 3365 | -0.474 | -0.3989 | No |
| 174 | ACSS1 |  |  | 3375 | -0.475 | -0.3976 | No |
| 175 | PCDH9 |  |  | 3422 | -0.486 | -0.4063 | Yes |
| 176 | JAG1 |  |  | 3424 | -0.486 | -0.4027 | Yes |
| 177 | EGFR |  |  | 3433 | -0.488 | -0.4010 | Yes |
| 178 | NPAS3 |  |  | 3434 | -0.488 | -0.3971 | Yes |
| 179 | RNF180 |  |  | 3455 | -0.494 | -0.3987 | Yes |
| 180 | OLIG1 |  |  | 3486 | -0.503 | -0.4029 | Yes |
| 181 | ZFP36L1 |  |  | 3487 | -0.503 | -0.3989 | Yes |
| 182 | GAS1 |  |  | 3494 | -0.505 | -0.3965 | Yes |
| 183 | TRIL |  |  | 3505 | -0.510 | -0.3952 | Yes |
| 184 | DIO2 |  |  | 3510 | -0.511 | -0.3922 | Yes |
| 185 | LRRC4C |  |  | 3512 | -0.512 | -0.3885 | Yes |
| 186 | SLC1A3 |  |  | 3516 | -0.513 | -0.3852 | Yes |
| 187 | TSPAN7 |  |  | 3528 | -0.520 | -0.3841 | Yes |
| 188 | TNK2 |  |  | 3539 | -0.524 | -0.3827 | Yes |
| 189 | BAALC |  |  | 3542 | -0.525 | -0.3790 | Yes |
| 190 | SOX9 |  |  | 3560 | -0.531 | -0.3795 | Yes |
| 191 | CACNB2 |  |  | 3564 | -0.532 | -0.3761 | Yes |
| 192 | PARP9 |  |  | 3571 | -0.536 | -0.3734 | Yes |
| 193 | SERPINE2 |  |  | 3573 | -0.537 | -0.3695 | Yes |
| 194 | LRP1 |  |  | 3584 | -0.540 | -0.3679 | Yes |
| 195 | RASSF2 |  |  | 3586 | -0.541 | -0.3639 | Yes |
| 196 | LAMB2 |  |  | 3588 | -0.542 | -0.3599 | Yes |
| 197 | LRRC17 |  |  | 3590 | -0.543 | -0.3558 | Yes |
| 198 | ILDR2 |  |  | 3592 | -0.544 | -0.3518 | Yes |
| 199 | DST |  |  | 3597 | -0.545 | -0.3486 | Yes |
| 200 | SOX2 |  |  | 3613 | -0.555 | -0.3483 | Yes |
| 201 | FOS |  |  | 3621 | -0.561 | -0.3457 | Yes |
| 202 | LSAMP |  |  | 3623 | -0.561 | -0.3415 | Yes |
| 203 | CD9 |  |  | 3634 | -0.567 | -0.3398 | Yes |
| 204 | DUSP6 |  |  | 3648 | -0.577 | -0.3387 | Yes |
| 205 | FGFBP3 |  |  | 3649 | -0.578 | -0.3341 | Yes |
| 206 | RFTN2 |  |  | 3654 | -0.582 | -0.3306 | Yes |
| 207 | MLC1 |  |  | 3659 | -0.584 | -0.3271 | Yes |
| 208 | NLGN4X |  |  | 3666 | -0.589 | -0.3241 | Yes |
| 209 | FAT1 |  |  | 3685 | -0.604 | -0.3242 | Yes |
| 210 | SEL1L3 |  |  | 3686 | -0.604 | -0.3194 | Yes |
| 211 | BMP7 |  |  | 3698 | -0.613 | -0.3175 | Yes |
| 212 | ZBTB20 |  |  | 3702 | -0.617 | -0.3134 | Yes |
| 213 | CLSTN1 |  |  | 3703 | -0.621 | -0.3085 | Yes |
| 214 | RORA |  |  | 3716 | -0.630 | -0.3068 | Yes |
| 215 | GNG7 |  |  | 3722 | -0.633 | -0.3031 | Yes |
| 216 | SLC35F1 |  |  | 3724 | -0.635 | -0.2984 | Yes |
| 217 | AIF1L |  |  | 3725 | -0.635 | -0.2933 | Yes |
| 218 | MEIS1 |  |  | 3727 | -0.637 | -0.2886 | Yes |
| 219 | STON2 |  |  | 3739 | -0.658 | -0.2863 | Yes |
| 220 | PCDH7 |  |  | 3745 | -0.665 | -0.2824 | Yes |
| 221 | TGFB2 |  |  | 3746 | -0.667 | -0.2771 | Yes |
| 222 | SYT11 |  |  | 3748 | -0.669 | -0.2721 | Yes |
| 223 | CADM4 |  |  | 3751 | -0.673 | -0.2673 | Yes |
| 224 | MMP16 |  |  | 3753 | -0.675 | -0.2623 | Yes |
| 225 | HSP90B1 |  |  | 3757 | -0.681 | -0.2577 | Yes |
| 226 | ITPR2 |  |  | 3758 | -0.682 | -0.2523 | Yes |
| 227 | FAM181B |  |  | 3760 | -0.686 | -0.2471 | Yes |
| 228 | F3 |  |  | 3764 | -0.693 | -0.2424 | Yes |
| 229 | LIMA1 |  |  | 3774 | -0.706 | -0.2393 | Yes |
| 230 | SALL3 |  |  | 3787 | -0.720 | -0.2368 | Yes |
| 231 | MAP3K1 |  |  | 3796 | -0.737 | -0.2332 | Yes |
| 232 | SOX8 |  |  | 3797 | -0.737 | -0.2273 | Yes |
| 233 | SSPN |  |  | 3808 | -0.756 | -0.2241 | Yes |
| 234 | NCAN |  |  | 3813 | -0.761 | -0.2191 | Yes |
| 235 | LRRN1 |  |  | 3816 | -0.766 | -0.2136 | Yes |
| 236 | TRIM9 |  |  | 3827 | -0.787 | -0.2101 | Yes |
| 237 | COL11A1 |  |  | 3828 | -0.788 | -0.2038 | Yes |
| 238 | WSCD1 |  |  | 3835 | -0.798 | -0.1991 | Yes |
| 239 | FJX1 |  |  | 3843 | -0.821 | -0.1945 | Yes |
| 240 | CLU |  |  | 3846 | -0.827 | -0.1885 | Yes |
| 241 | SLC7A11 |  |  | 3847 | -0.836 | -0.1819 | Yes |
| 242 | S100B |  |  | 3853 | -0.845 | -0.1765 | Yes |
| 243 | LRRN3 |  |  | 3859 | -0.856 | -0.1711 | Yes |
| 244 | COL6A1 |  |  | 3863 | -0.872 | -0.1650 | Yes |
| 245 | FABP7 |  |  | 3867 | -0.888 | -0.1588 | Yes |
| 246 | SCRG1 |  |  | 3869 | -0.904 | -0.1519 | Yes |
| 247 | TNC |  |  | 3874 | -0.951 | -0.1455 | Yes |
| 248 | PMP2 |  |  | 3876 | -0.959 | -0.1381 | Yes |
| 249 | SLC1A2 |  |  | 3877 | -0.969 | -0.1304 | Yes |
| 250 | C1orf61 |  |  | 3879 | -0.986 | -0.1229 | Yes |
| 251 | NEAT1 |  |  | 3880 | -1.005 | -0.1149 | Yes |
| 252 | PTCH1 |  |  | 3883 | -1.016 | -0.1074 | Yes |
| 253 | PTN |  |  | 3884 | -1.022 | -0.0993 | Yes |
| 254 | NPNT |  |  | 3886 | -1.025 | -0.0915 | Yes |
| 255 | CYR61 |  |  | 3887 | -1.037 | -0.0832 | Yes |
| 256 | GPM6B |  |  | 3889 | -1.047 | -0.0752 | Yes |
| 257 | CXCL14 |  |  | 3892 | -1.060 | -0.0673 | Yes |
| 258 | ID4 |  |  | 3893 | -1.061 | -0.0589 | Yes |
| 259 | IGFBP5 |  |  | 3895 | -1.082 | -0.0506 | Yes |
| 260 | GFAP |  |  | 3907 | -1.280 | -0.0435 | Yes |
| 261 | PTPRZ1 |  |  | 3913 | -1.342 | -0.0342 | Yes |
| 262 | IGFBP2 |  |  | 3917 | -1.404 | -0.0239 | Yes |
| 263 | MALAT1 |  |  | 3924 | -1.583 | -0.0130 | Yes |
| 264 | PDGFRA |  |  | 3928 | -1.773 | 0.0003 | Yes |
Table: GSEA details [plain text format]

  

Fig 2: RICHARDS\_NOWAKOWSKI\_SCIENCE\_2017\_ASTROCYTE\_UPREG: Random ES distribution      
 Gene set null distribution of ES for **RICHARDS\_NOWAKOWSKI\_SCIENCE\_2017\_ASTROCYTE\_UPREG**
